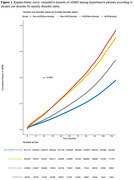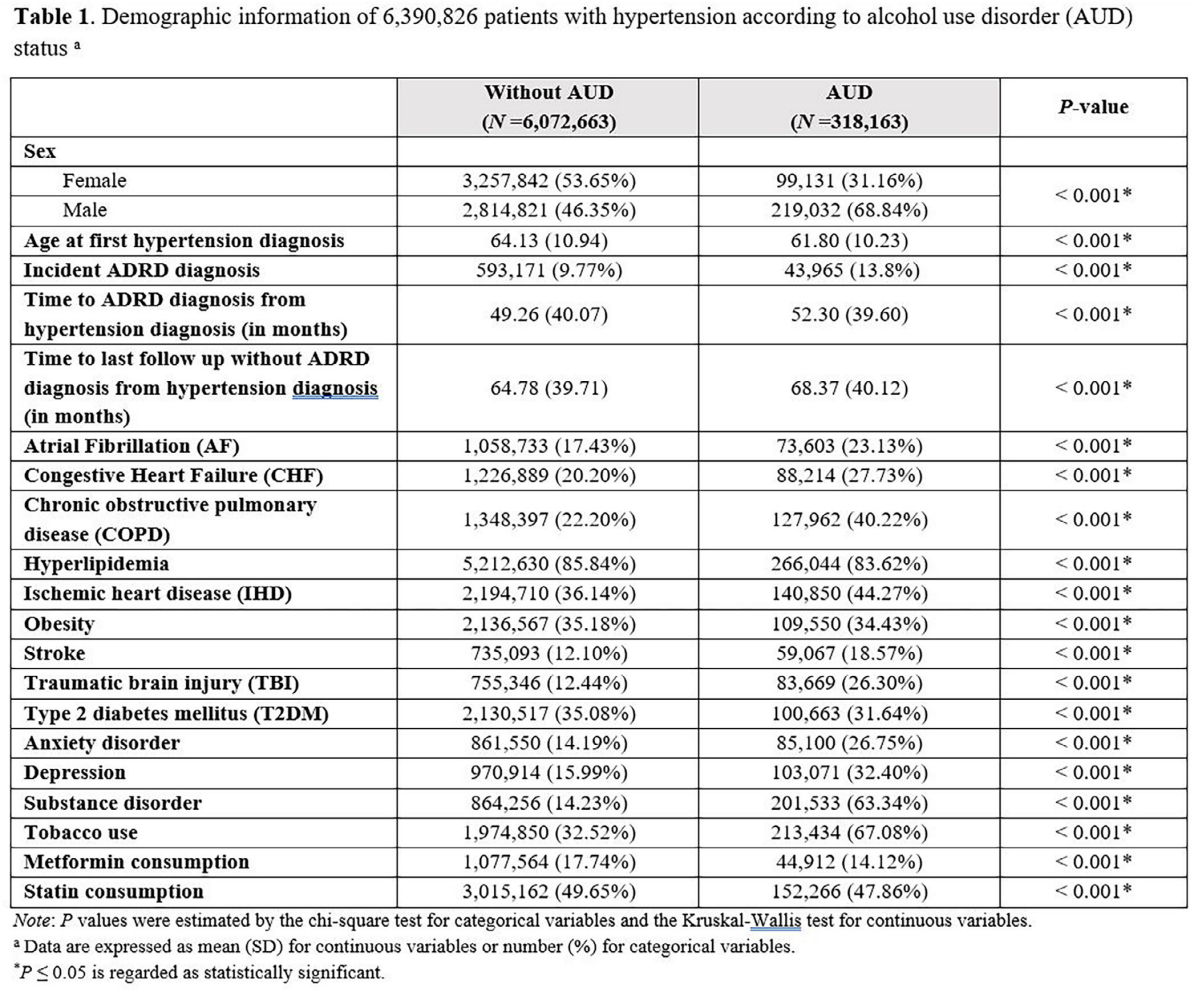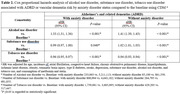# Additive and interactive roles of alcohol use disorder and anxiety disorder on higher risk of Alzheimer’s disease and related dementias amongst hypertensive patients in high‐volume claims data: a retrospective study

**DOI:** 10.1002/alz.084818

**Published:** 2025-01-09

**Authors:** Sori Kim Lundin, Xinyue Hu, Jingna Feng, Yong Chen, Paul E Schulz, Cui Tao

**Affiliations:** ^1^ Center for Biomedical Semantics and Data Intelligence (CBSDI), University of Texas Health Science Center at Houston, Houston, TX USA; ^2^ School of Public Health, The University of Texas Health Science Center at Houston, Houston, TX USA; ^3^ School of Biomedical Informatics, The University of Texas Health Science Center at Houston, Houston, TX USA; ^4^ Perelman School of Medicine, University of Pennsylvania, Philadelphia, PA USA; ^5^ John P. and Kathrine G. McGovern Medical School at UTHealth, Houston, TX USA; ^6^ McWilliams School of Biomedical Informatics, The University of Texas Health Science Center at Houston, Houston, TX USA

## Abstract

**Background:**

Alcohol use disorder (AUD) among elderly population is a strong risk factor for dementia. Anxiety disorder also poses a great toll on cognitive health and is commonly diagnosed among individuals with AUD. However, the additive and interactive roles of AUD‐anxiety disorder comorbidities on cognitive disorders such as Alzheimer’s disease and related dementias (ADRD) is poorly studied.

**Method:**

6,390,826 individuals with hypertension were selected from US‐based Optum de‐identified Clinformatics® Data Mart. Baseline characteristics were compared between groups by the Chi‐square test for categorical variables and the Kruskal‐Wallis test for continuous variables. Kaplan‐Meier and adjusted cox proportional hazards (PH) model were used to study the additive and interactive associations of AUD and anxiety disorder in relation to the time from hypertension to ADRD.

**Result:**

318,163 individuals with AUD and 6,072,663 without AUD were identified. Almost 70% of the AUD patients were male. Incidence of anxiety disorder, depression, substance use disorder, tobacco use, and COPD were significantly higher in AUD patients compared to non‐AUD patients (all *P* < 0.0001). In the Kaplan‐Meier plot, hypertensive individuals with AUD‐anxiety disorder comorbidity showed the highest cumulative hazards of ADRD, followed by those with anxiety disorder but no AUD. Individuals without AUD and anxiety disorder had the least cumulative hazards of ADRD. These four groups had a significant difference in their risk of ADRD (*P* < 0.0001). Using Cox PH analyses with additive terms, individuals with AUD comorbidity showed a 33% and 41% higher adjusted hazard ratio (aHR) of ADRD development than those without, among patients with and without anxiety disorder, respectively. Interactive terms of AUD and anxiety disorder were associated with 3% increase in aHR (*P*=0.012).

**Conclusion:**

In conclusion, our study found that AUD‐anxiety disorder comorbidities were associated with a higher risk of ADRDs from large‐volume claims data. We also found that the interaction of AUD‐anxiety disorders was linked to harmful effects against ADRD development. These results strongly suggest a critical need for combining medical and psychological efforts to fight dementia through integrative intervention on AUD and anxiety disorder.